# Supplementary material for: Patched and Costal-2 mutations lead to differences in tissue overgrowth autonomy
Source: Fly (Austin). 2022 Apr 25;16(1):176–89. doi: 10.1080/19336934.2022.2062991 (PMC9045829; doi:10.1080/19336934.2022.2062991)
Supplement: Supplemental Material [file KFLY_A_2062991_SM8566.docx]

**Supplemental Figure 1**

**
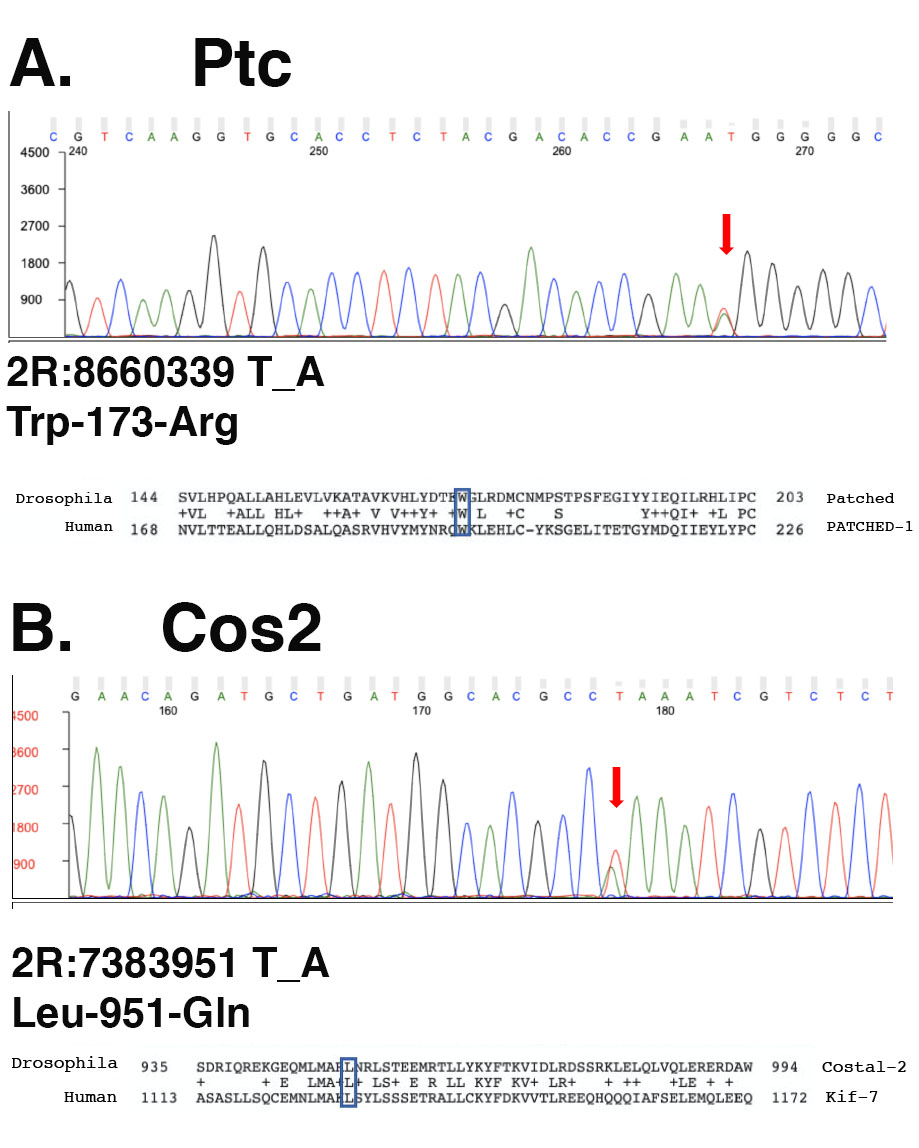
**

**Supplemental Figure 1: *Ptc^B.2.13^* and *Cos2^F.1.4^* have missense mutations in conserved amino acids**

*Ptc* and *Cos2* heterozygous animals were sequenced via Sanger sequencing. (A) *Ptc^B.2.13^* mutation resides at 2R:8660339 resulting in a Trp-Arg mutation that is in a residue conserved between *Patched* and *PATCHED1.* (B). *Cos2^F.1.4^* mutation resides at 2R:7383951 resulting in a Leu-Gln mutation in a conserved residue between *Costal2* and *Kif7*.

**Supplemental Figure 2**


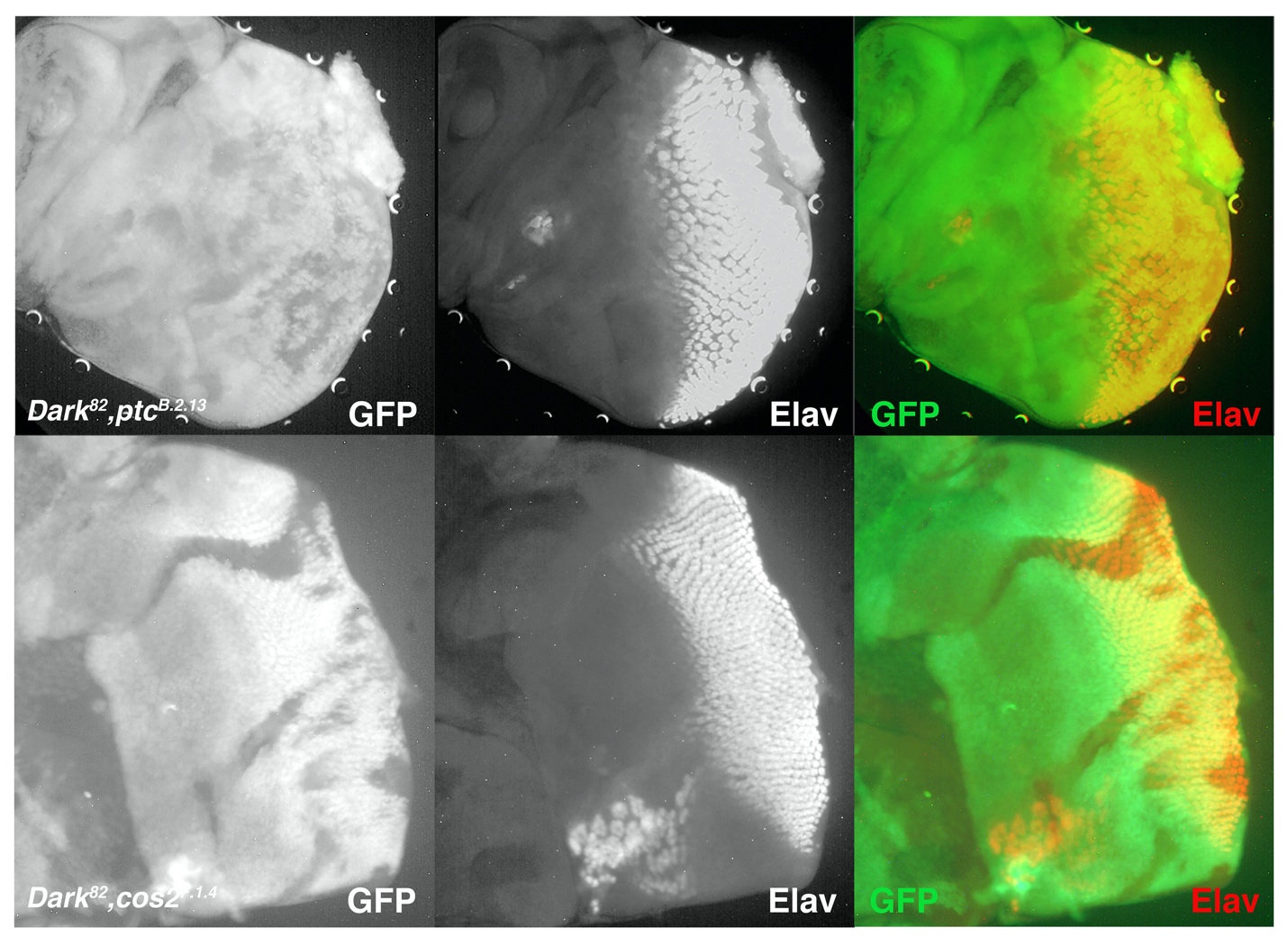


**Supplemental Figure 2: *Ptc^B.2.13^* and *Cos2^F.1.4^* clones result in premature Elav expression and eye differentiation.**

Elav levels visualized in third instar imaginal eye discs through staining and fluorescent microscopy for crosses of *Ey-Flp;FRT42D, ubi-GFP* mated top: *FRT42D, Ptc^B.2.13^, Dark^82^* or bottom *FRT42D, Cos2^F.1.4^, Dark^82^* (mutant tissue is GFP negative). In both mutants ectopic Elav expression can be seen in mutant clones prior to the wave of differentiation seen in the rest of the eye.


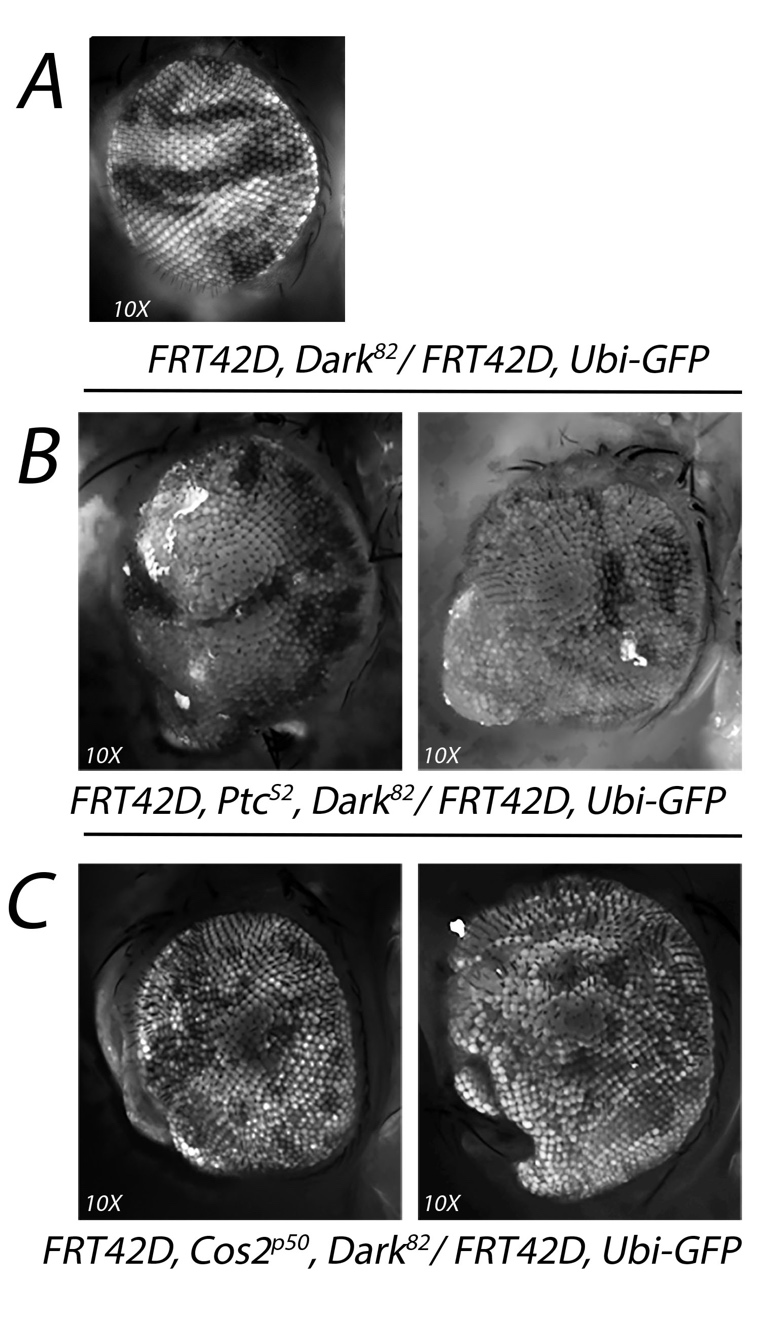
**Supplemental Figure 3**

**Supplemental Figure 3: Independently generated alleles of *Ptc* and *Cos2* lead to differences in overgrowth autonomy.**

Fluorescent apotome images to visualize ommatidial organization by mating *Ey-Flp; FRT42D, ubi-GFP* to (A) control eye *FRT42D, Dark^82^* (B) *FRT42D, ptc^S2^, Dark^82^* (F) *FRT42D, Cos2^p50^, Dark^82^* (mutant tissue, darker, fluorescent negative in all panels, adjacent wild type tissue brighter, GFP positive in all panels). All images were visualized at 10x magnification; all flies are oriented anterior to the left.

**Supplemental Figure 4**

**
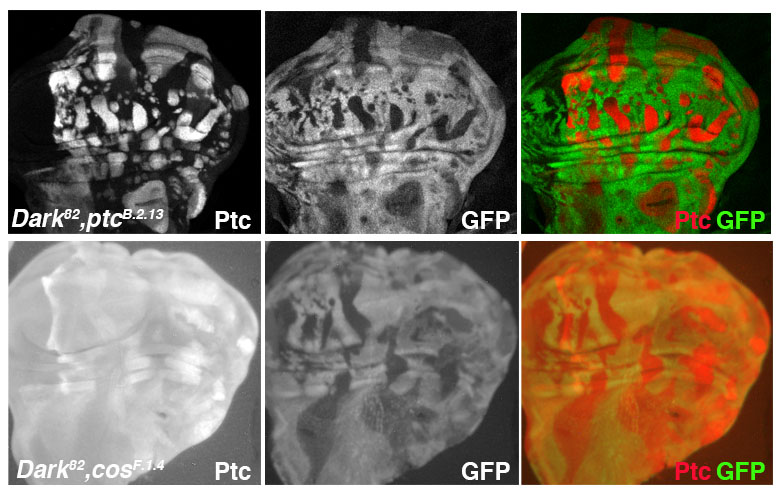
**

**Supplemental Figure 4: *Ptc^B.2.13^* and *Cos2^F.1.4^* autonomously up-regulated Ptc within mutant clones in the anterior compartment of imaginal wing discs**

Ptc levels visualized in third instar imaginal wing discs through staining and fluorescent microscopy for crosses of *UBX-Flp;FRT42D, ubi-GFP* mated top: *FRT42D, ptc^B.2.13^, Dark^82^* or bottom *FRT42D, Cos2^F.1.4^, Dark^82^* (mutant tissue is GFP negative). Anterior portion of wing discs are oriented to the right.

**Supplemental Figure 5**

**
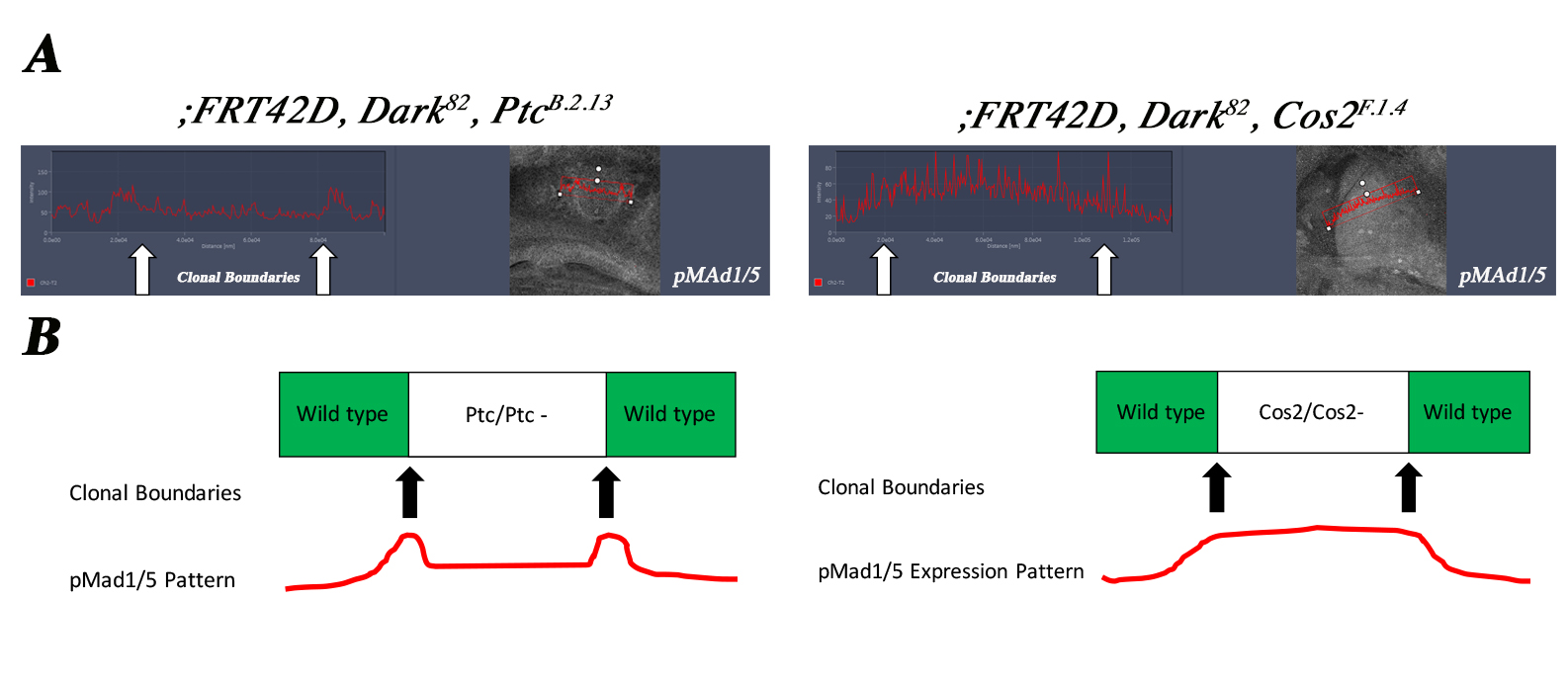
­­**

**Supplemental Figure 5:** Deregulation patterns of pMad in *Ptc* and *Cos2* mosaic imaginal discs. *Ptc^B.2.13^* mutant clones result in peaks of pMad deregulation immediately outside of the mutant clones. The *Ptc/Ptc-* mutant clones have reduced expression patterns of pMad. In contrast, *Cos2^F.1.4^* clones result in an autonomous increase of pMad, the deregulation of pMad extends into the adjacent wild type tissue as well resulting in the autonomous and non-autonomous over expression observed in *Cos2/Cos2-* clones.
